# Risk Factor Analyses for Immune Reconstitution Inflammatory Syndrome in a Randomized Study of Early vs. Deferred ART during an Opportunistic Infection

**DOI:** 10.1371/journal.pone.0011416

**Published:** 2010-07-01

**Authors:** Philip M. Grant, Lauren Komarow, Janet Andersen, Irini Sereti, Savita Pahwa, Michael M. Lederman, Joseph Eron, Ian Sanne, William Powderly, Evelyn Hogg, Carol Suckow, Andrew Zolopa

**Affiliations:** 1 Stanford University, Palo Alto, California, United States of America; 2 Statistical and Data Analysis Center, Harvard School of Public Health, Boston, Massachusetts, United States of America; 3 National Institute for Allergy and Infectious Diseases, National Institutes of Health, Bethesda, Maryland, United States of America; 4 University of Miami, Miami, Florida, United States of America; 5 Case Western University, Cleveland, Ohio, United States of America; 6 University of North Carolina, Chapel Hill, North Carolina, United States of America; 7 Wits Health Consortium, Helen Joseph Hospital, Johannesburg, South Africa; 8 University College Dublin, Dublin, Ireland; 9 Social and Scientific Systems, Incorporated, Silver Spring, Maryland, United States of America; 10 Frontier Science and Technology Research Foundation, Amherst, New York, United States of America; Instituto de Pesquisa Clinica Evandro Chagas, Brazil

## Abstract

**Background:**

Immune reconstitution inflammatory syndrome (IRIS) is reported widely in patients initiating antiretroviral therapy (ART). However, few studies are prospective, and no study has evaluated the impact of the timing of ART when allocated randomly during an acute opportunistic infection (OI).

**Methodology/Principal Findings:**

A5164 randomized 282 subjects with AIDS-related OIs (tuberculosis excluded), to early or deferred ART. IRIS was identified prospectively using pre-defined criteria. We evaluated associations between IRIS and baseline variables in subjects with follow-up on ART using Wilcoxon and Fisher's exact tests, logistic regression, and Cox models with time-varying covariates. Twenty of 262 (7.6%) subjects developed IRIS after a median of 33 days on ART. Subjects with fungal infections (other than pneumocystis) developed IRIS somewhat more frequently (OR = 2.7; 95% CI: 1.02, 7.2; p-value = 0.06 (using Fisher's exact test)). In Cox models, lower baseline and higher on-treatment CD4+ T-cell counts and percentage were associated with IRIS. Additionally, higher baseline and lower on-treatment HIV RNA levels were associated with IRIS. Corticosteroids during OI management and the timing of ART were not associated with the development of IRIS.

**Implications:**

In patients with advanced immunosuppression and non-tuberculous OIs, the presence of a fungal infection, lower CD4+ T-cell counts and higher HIV RNA levels at baseline, and higher CD4+ T-cell counts and lower HIV RNA levels on treatment are associated with IRIS. Early initiation of ART does not increase the incidence of IRIS, and concern about IRIS should not prompt deferral of ART.

**Trial Registration:**

ClinicalTrials.gov NCT00055120

## Introduction

Potent combination antiretroviral therapy (ART) has dramatically reduced morbidity and mortality associated with HIV infection [1], [2], but its use can be complicated by immune reconstitution inflammatory syndrome (IRIS) [3]. Although no uniform definition exists, the diagnosis of IRIS requires the worsening of a recognized (“paradoxical” IRIS) or unrecognized (“unmasking” IRIS) pre-existing infection in the setting of improving immunologic function. The pathophysiology is not well-defined, but the prevailing view is that IRIS reflects the restoration of pathogen-specific immune response to microbial antigens [4].

IRIS has been reported in 10–40% of patients initiating ART [5], [6], [7], [8]. In some reports, IRIS has resulted in increased hospitalizations [6] but generally does not portend a poor long-term prognosis [9]. Most studies of IRIS in patients initiating ART with active opportunistic infections (OIs) have been retrospective and focused on tuberculosis, cryptococcosis, and *Mycobacterium avium* complex (MAC) infection [5], [6]. Although there have been two recent prospective cohort studies evaluating the risk factors for cryptococcal IRIS in resource-limited settings [10], [11], there are no IRIS risk factor analyses where the impact of the timing of ART has been evaluated in a randomized clinical trial.

Previously reported risk factors for the development of IRIS include low baseline CD4+ T-cell count, a robust immunologic and virologic response to ART, and a short interval between initiation of treatment for the OI and ART [5], [6], [12]. Here, we report a risk factor analysis for IRIS during a randomized clinical trial of early versus deferred ART in the setting of an acute OI [13].

## Results

Of the 282 subjects enrolled in the trial, 262 initiated ART, had at least one subsequent study visit and were included in this analysis. The median age was 38 years and 86% were men. Thirty percent (78/262) of individuals were African American, 35% (91/262) were Hispanic, and 7% (18/262) were from South Africa. The median CD4+ T-cell count was 29 cells/µL [IQR 12, 55] with a median plasma HIV RNA level of 5.0 log_10_ copies/ml [IQR 4.8, 5.7]. Prior to ART initiation, 65% (171/262) of subjects were diagnosed with PCP (100 confirmed and 71 presumptive cases), 14% (37/262) with cryptococcosis, 14% (37/262) with bacterial infections, 6% (16/262) with mycobacterial infections, 5% (14/262) with toxoplasmosis, and 4% (10/262) with histoplasmosis. Fifty-three percent of subjects (138/262) were diagnosed with more than one OI at baseline. Ninety-two percent of subjects (241/262) were ART-naïve at study entry.

Twenty of 262 subjects (7.6%; 95% CI: 4.7%–11.5%) had a confirmed IRIS diagnosis by a study chair (Table 1). Three cases of potential IRIS did not meet study definition of IRIS after review. Two potential cases of IRIS with dermatologic manifestations were excluded as their presentations were not deemed specific enough to meet the study definition for IRIS. The other potential case was excluded when an alternative diagnosis became more likely after the initial report was filed. Review of study subjects who had received corticosteroids or NSAIDs did not reveal additional cases of IRIS. IRIS was confirmed in 8% of subjects (14/171) with PCP, 5% (2/37) with bacterial infections, 15% (7/47) with non-PCP fungal infections (14% of subjects with cryptococcosis and 20% of subjects with histoplasmosis), and 13% of subjects (2/16) with mycobacterial infections.

**Table 1:** Characteristics of IRIS subjects^1^.

Gender, age(yrs)	OIs prior to ART initiation	IRIS etiology	IRIS symptoms	Days between OI treatment and ART initiation	Days between ART initiation and IRIS diagnosis	Baseline CD4+ count→IRIS CD4+ count (cells/µL)	Baseline HIV RNA→IRIS HIV RNA (log_10_ copies/mL)
M, 45	CM, PCP	CM^2^	Headache	1 (CM), 10 (PCP)	62	10→80	4.7→2.6
M, 44	MAC, PCP	MAC^2^	Fever, lymphadenopathy	6 (MAC), 43 (PCP)	26	62→759^4^	5.5→3.4
M, 34	PCP	Cryptococcus^3^	Headache, submandibular mass	7	117	1→66	4.7→1.9
F, 47	PCP	TB^3^	Fever, cough, pleuritic chest pain	8	34	64→102	5.0→3.9
M, 40	PCP	PCP^2^	Fever, cough, dyspnea	9	82	25→186	6.1→3.0
M, 45	PCP, Candida esophagitis	VZV^3^	Vesicular rash	9	138	5→153	6.0→2.0
M, 30	PCP	CMV^3^	Eye redness, visual loss	11	43	17→144	5.7→2.7
M, 42	PCP, PNA	PCP^2^	Fever, cough, dyspnea	11	22	18→47	4.7→2.7
M, 38	Histoplasmosis	HCV^3^	Nausea, vomiting, hepatitis	14	229	3→37	6.1→6.0^5^
M, 31	MAC, CM, PNA	MAC^2^	Fever	23 (MAC), 48 (CM)	31	44→74	5.7→2.9
M, 39	CM	CM^2^	Fever, headache	29	26	29→80	4.7→2.5
M, 42	CM	CM^2^	Headache, nuchal rigidity, photophobia	37	116	31→128	4.8→2.2
M, 37	PCP	PCP^2^	Fever, cough	42	35	76→481	6.3→3.6
M, 27	PCP, Candida esophagitis	MAC^3^	Fever, night sweats, diarrhea	44	15	52→43	6.3→5.7
M, 42	PCP, KS	MAC^3^	Fever, night sweats, lymphadenopathy	45	50	14→231	5.7→2.4
F, 35	PCP	MAC^3^	Fever, night sweats	46	28	17→464	6.0→2.1
M, 45	Histoplasmosis, Cryptosporidia	Histoplasmosis^2^	Fever	47 (Histoplasma), 42 (Cryptosporidia)	18	21→190^4^	4.9→3.2
M, 24	CM	CM^2^	Blurry vision (due to papilledema)	48	29	14→44	4.7→2.3
M, 45	PCP	PCP^2^	Fever, chills, dyspnea	49	13	22→101^4^	4.6→1.9
M, 36	PCP	MAC^3^	Fever, lymphadenopathy	54	29	40→357	5.4→2.6

1 IRIS = immune reconstitution inflammatory syndrome; OI = opportunistic infection; ART = combination antiretroviral therapy; M = male; F = female; CM = cryptococcal meningitis; PCP = *Pneumocystis jirovecii* pneumonia; PNA = bacterial pneumonia; MAC = *Mycobacterium avium* complex; TB = tuberculosis; VZV = varicella-zoster virus; CMV = cytomegalovirus; HCV = hepatitis C virus; KS = Kaposi's sarcoma.

2 = Paradoxical IRIS.

3 = Unmasking IRIS.

4 IRIS CD4+ T-cell counts listed in table, in general, are from at/or before time of IRIS diagnosis; however, these values are from after time of IRIS but all within 3 days of time of IRIS).

5 = at the time of IRIS diagnosis (based on liver biopsy findings), ART had been discontinued; previously subject had robust response to ART with HIV RNA levels below limit of quantification.

The median time to IRIS diagnosis after ART initiation was 33 days [IQR 26, 72] and was not significantly different between those assigned to early and deferred ART (72 vs. 29 days, respectively; p = 0.25). At the time of IRIS diagnosis, the median change in CD4+ T cells was an increase of 88 cells/µL [IQR 36, 193] and the median decrease in HIV RNA levels was 2.7 log_10_ copies/mL [IQR 2.0, 2.9]. Eleven subjects had paradoxical IRIS and 9 subjects had unmasking IRIS. The median time to IRIS diagnosis after ART initiation and the median change in CD4+ T cells and HIV RNA levels at the time of IRIS were similar between those subjects who developed paradoxical and unmasking IRIS (data not shown). Six subjects had IRIS reactions to MAC, 5 to cryptococcus, 4 to *Pneumocystis jirovecii*, and 1 each to *Mycobacterium tuberculosis*, varicella-zoster virus, cytolomegalovirus, hepatitis C virus, and *Histoplasma capsulatum*. The presentations of IRIS are described in Table 1.

Eighteen of twenty subjects continued ART after the diagnosis of IRIS. Corticosteroids were used in the treatment of 35% (7/20) of IRIS cases for a median of 83 days and for 3 of the 5 cases of cryptococcal IRIS. The majority of IRIS cases resolved quickly and without sequelae. However, the four most protracted cases (one in the early ART treatment arm and three in the deferred ART arm) were due to cryptococcus. Symptoms persisted to weeks 24, 27, 31, and 46 of ART before finally resolving. However, the 48 week study outcomes of these cases were favorable with no additional OIs occurring, and all surviving to study completion. Only one of the 20 subjects with IRIS died (comparable to the 8.5% mortality in the entire ACTG A5164 study population), and this death was not due to IRIS, as determined by the local investigator and confirmed by the study chairs.

In univariate analyses, no baseline variables, other than entry OI, were significantly associated with the development of IRIS (Table 2). Importantly, subjects assigned to early ART were no more likely to develop IRIS than those assigned to deferred ART (OR = 0.60; 95% CI: 0.24, 1.5; p = 0.35). Subjects with non-PCP fungal infections developed IRIS somewhat more frequently (OR = 2.7; 95% CI: 1.02, 7.2; p = 0.06 (Fisher's exact test); p = 0.045 (non-exact test)). Lower baseline CD4+ T-cell count or percentage were not associated with the development of IRIS, nor were markers of OI disease severity such as hospitalization at study entry or elevated LDH. The use of corticosteroids during the management of the acute OI was not associated with a significant reduction in the frequency of IRIS (OR = 0.57; 95% CI: 0.23, 1.4; p = 0.24), although no subjects developed IRIS while still on corticosteroids.

**Table 2:** Univariate Analyses of Predictors for IRIS.

Characteristic	Subjects with IRIS	Subjects without IRIS	Odds Ratio (95% CI)	P-value^1^
Total	20	242		
Median Age (IQR)	40 (35, 45)	38 (33, 44)	N.A.	0.91
Ethnicity				
Black	5 (25%)	73(30%)	Ref	0.58
Hispanic	10 (50%)	81 (33%)	1.80 (0.59–5.52)	
Other	4 (20%)	71 (29%)	0.82 (0.21–3.19)	
South Africa	1 (5%)	17 (7%)	0.86 (0.09–7.84)	
Treatment Arm				
Early	8 (40%)	127 (52%)	0.60 (0.24–1.53)	0.35
Deferred	12 (60%)	115 (48%)		
ART prior to entry				
Naïve	18 (90%)	223 (92%)	0.77 (0.17–3.55)	0.67
Experienced	2 (10%)	19 (8%)		
ART Regimen				
PI-based	17 (85%)	210 (87%)	0.86 (0.24–3.11)	0.74
Non-PI-based	3 (15%)	32 (13%)		
Hospitalized at ART Start				
Yes	4 (20%)	33 (14%)	1.58 (0.50–5.03)	0.21
No	16 (80%)	209 (86%)		
Steroids during Acute OI				
Yes	9 (45%)	141 (58%)	0.59 (0.23–1.47)	0.35
No	11 (55%)	101 (42%)		
Pre-ART OIs^2^				
PCP	14 (70%)	157 (65%)	1.26 (0.47–3.41)	0.81
Bacterial Infection	2 (10%)	35 (14%)	0.66 (0.15–2.96)	0.75
Mycobacterial Infection	2 (10%)	14 (6%)	1.81 (0.38–8.59)	0.35
Non-PCP Fungal Infection	7 (35%)	40 (17%)	2.72 (1.02–7.24)	0.06^3^
Toxoplasmosis	0 (0%)	14 (6%)	N.A.^4^	0.61
Median Baseline CD4+ (cells/µL) (IQR)	22 (14, 42)	31 (12, 56)	N.A.	0.30
Median Baseline CD4+% (IQR)	3.5 (1, 7)	4 (2, 7)	N.A.	0.66
Median Baseline CD8+ (cells/µL) (IQR)	472 (217, 793)	446 (270, 719)	N.A.	0.97
Median Baseline Viral Load (log_10_ copies/mL) (IQR)	5.5 (4.8, 6.0)	5.0 (4.8, 5.7)	N.A.	0.28

1 P-values by Fisher's exact test for dichotomous variables and Wilcoxon Rank Sum Test for continuous variables.

2 Participants could have mulitiple opportunistic infections (OIs) so percents do not sum to 1.

3 At margin of significance; P-value is 0.045 using non-exact test.

4 Not calculated due to cell containing 0.

N.A. = Not applicable.

Most subjects in the study population had a robust response to ART. The median 4-week change in CD4+ T-cell count and plasma HIV RNA level was +71 cells/µL [IQR 30, 141] and −2.10 log_10_ copies/mL [IQR −2.60, −1.55]. Table 3 groups subjects into 3 groups those who developed IRIS by week 4 (n = 10), those who developed IRIS after week 4 (n = 10), and subjects who never developed IRIS (n = 242). At week 4 of ART, subjects already diagnosed with IRIS had a median CD4+ T-cell count of 171 cells/µL (IQR 74, 357), while subjects who would later have IRIS diagnosed had a median CD4+ T-cell count of 91 cells/µL (IQR 75, 144) and subjects who never had IRIS had a median CD4+ T-cell count of 116 cells/µL (IQR 56, 192). Subjects who developed IRIS by 4 weeks also had a higher median CD4+ percentage (13.0 [IQR 7.0, 18.0] vs. IRIS post-week 4: 8.5 [IQR 7.0, 12.0] vs. No IRIS: 8.0 [5.0, 13.0]) and lower median log_10_ HIV RNA levels (2.6 [IQR 2.4, 2.9] vs. IRIS post-week 4: 3.2 [IQR 2.5, 3.6] vs. No IRIS: 3.1 [IQR 2.6, 3.6]). Grouping the subjects into those who developed IRIS by week 8 (n = 14), those who developed IRIS after week 8 (n = 6), and subjects who never developed IRIS (n = 242) showed a similar pattern with subjects who developed IRIS before this time period showing a more robust immunologic and virologic response compared to those who developed IRIS subsequently and those who never developed IRIS (data not shown).

**Table 3:** T-cell subsets and HIV RNA Levels versus IRIS.

Change from Baseline	IRIS		Baseline		Week 4
		n	Median (IQR)	n	Median (IQR)
CD4+ (cells/µL)					
	≤Wk 4	10	26 (18, 44)	10	171 (74, 357)
	>Wk 4	10	16 (5, 31)	10	91 (75, 144)
	No IRIS	242	31 (12, 56)	225	116 (56, 192)
CD4+%					
	≤Wk 4	10	5.5 (2.0, 7.0)	10	13.0 (7.0, 18.0)
	>Wk 4	10	3.0 (1.0, 6.0)	10	8.5 (7.0, 12.0)
	No IRIS	239	4.0 (2.0, 7.0)	225	8.0 (5.0, 13.0)
CD8+ (cells/µL)					
	≤Wk 4	10	362 (198, 585)	10	692 (507, 1772)
	>Wk 4	10	697 (269, 823)	10	613 (477, 1273)
	No IRIS	238	446 (270, 719)	225	770 (488, 1183)
CD8+%					
	≤Wk 4	10	54 (45, 63)	10	56 (44, 65)
	>Wk 4	10	70 (58, 74)	10	61 (53, 68)
	No IRIS	238	62 (49, 73)	225	62 (52, 70)
log_10_ viral load					
	≤Wk 4	10	5.2 (4.7, 5.7)	10	2.6 (2.4, 2.9)
	>Wk 4	10	5.7 (4.8, 6.1)	10	3.2 (2.5, 3.6)
	No IRIS	242	5.0 (4.7, 5.7)	229	3.1 (2.6, 3.6)

1 IRIS = immune reconstitution inflammatory syndrome; 2 ART = combination antiretroviral therapy.

In multivariate Cox models using time-varying covariates, lower baseline CD4+ T-cell count (HR = 0.79 per 10 additional CD4+ cells/µL; 95% CI: 0.65, 0.97; p = 0.022), higher CD4+ T-cell counts on ART (HR = 1.08 per additional 10 CD4+ cells/µL; 95% CI: 1.03, 1.13; p = 0.002), and the presence of a baseline non-PCP fungal infection (HR = 3.01; 95% CI: 1.16, 7.80; p = 0.023) were significantly associated with the development of IRIS (Table 4). Lower baseline CD4+ percentage (HR = 0.17 per additional 10 increase in CD4%; 95% CI: 0.04, 0.69; p<0.001), higher CD4+ percentage on ART (HR = 3.90 per additional 10 increase in CD4%; 95% CI: 1.79, 8.47; p = 0.012) and baseline non-PCP fungal infection (HR = 3.08; 95% CI:1.20, 7.89; p = 0.019) were also significantly associated with IRIS.

**Table 4:** Cox Proportional Hazards Models Using Time Varying Covariate Models.

		ART start value	Covariate on ART	Non-PCP Fungal Infection
CD4+ (cells/µL)	Hazard Ratio (per additional 10 cells)	0.79	1.08	3.01
	95% Confidence Interval	0.65, 0.97	1.03, 1.13	1.16, 7.80
	P-value	0.022	0.002	0.023
CD4+%	Hazard Ratio (per additional 10%)	0.17	3.90	3.08
	95% Confidence Interval	0.04, 0.69	1.79, 8.47	1.20, 7.89
	P-value	<0.001	0.012	0.019
CD8+ (cells/µL)	Hazard Ratio (per additional 100 cells)	0.99	1.03	2.76
	95% Confidence Interval	0.93, 1.05	0.99, 1.07	1.09, 6.99
	P-value	0.66	0.21	0.032
log_10_ viral load	Hazard Ratio (per 1 log_10_ increase)	2.49	0.43	3.03
	95% Confidence Interval	1.19, 5.21	0.24, 0.78	1.20, 7.64
	P-value	0.015	0.006	0.019

Cox models evaluating change in CD4+ T-cells and CD4+ percentage (rather than the absolute values on ART) versus the risk of IRIS produced similar results. Low baseline CD4+ T-cell count (HR = 0.83 per additional 10 CD4+ cells/µL; 95% CI: 0.70, 1.00; p = 0.044), change in CD4+ T-cell count (HR = 1.08 per additional 10 CD4+ cells/µL; 95% CI: 1.03, 1.13; p = 0.002), and non-PCP fungal infection (HR = 3.03; 95% CI: 1.17, 7.84; p = 0.022) were significantly associated with the development of IRIS. Larger change in CD4+ percentage (HR = 3.52 per additional 10 increase in CD4%; 95% CI: 1.50, 8.27 ; p = 0.004) and non-PCP fungal infections (HR = 2.87; 95% CI 1.13, 7.29; p = 0.026) were both significantly associated with the development of IRIS, while low baseline CD4+ percentage did not reach significance in this model (HR = 0.36 per additional 10 increase in CD4%; 95% CI: 0.11, 1.16; p = 0.086).

In multivariate Cox models, baseline HIV RNA levels (HR = 2.49 per 1 log increase in HIV RNA; 95% CI:1.19, 5.21; p = 0.015), HIV RNA levels on ART (HR = 0.43 per 1 log increase in HIV RNA; 95% CI:0.24, 0.78; p = 0.006), and non-PCP fungal infections (HR = 3.03; 95% CI:1.20, 7.64; p = 0.019) also predicted the development of IRIS. There was no relationship between CD8+ T-cell counts and IRIS.

## Discussion

In this prospective study of risk factors associated with IRIS during a randomized clinical trial, the incidence of IRIS was 7.6% (95% CI: 4.7%–11.5%) over 48 weeks among those with follow-up on ART. Our estimate is in line with a prospective study in asymptomatic patients from South Africa starting ART which reported a rate of 10.4% [8]. Prospective studies of IRIS in patients with cryptococcal disease in South Africa and Thailand showed incidences of 17% and 13%, respectively [10], [11], which is in the same range as the 14% of subjects who developed IRIS with cryptococcal infections in our study. Our risk estimate of IRIS for subjects initiating ART with non-tuberculous mycobacterial disease is 13%. Importantly, these estimates are substantially lower than those from retrospective studies. For instance, in a retrospective study among patients initiating ART after tuberculosis, MAC or cryptococcal infections, 32% of patients developed IRIS [6]. Even in asymptomatic patients initiating ART, retrospective studies frequently report rates of IRIS in excess of 20% [7], [14].

The higher rates of IRIS reported in retrospective studies likely reflect differences in case definitions of IRIS, the effect of active case finding using retrospective data (case finding bias), lack of uniform diagnostic testing at suspected IRIS, and different study populations. While some cases of IRIS are straightforward (e.g., MAC lymphadenitis in a patient with immune recovery), other cases are less definitive (e.g., the development of genital herpes in a patient responding to ART). As this study was prospective, our results are more reflective of how the diagnosis of IRIS is made in practice. Less prevalent in this series are IRIS cases with primarily dermatologic manifestations which constitute the majority of cases in some retrospective studies [7], [12]. These can be difficult to distinguish from coincidental instances of dermatologic disease that are common in patients with advanced immune deficiency [15]. Validated and widely accepted disease-specific definitions of IRIS would help standardize reporting of IRIS. Efforts have been made to standardize definitions for TB IRIS and cryptococcal IRIS in recent years [16], and these efforts should be extended to standardize case definitions of IRIS for other OIs as well.

Initiation of ART closer to the diagnosis of the OI has been associated with the development of IRIS in at least two retrospective studies [6], [17]. Treatment guidelines cite the risk of IRIS as a potential downside to initiation of ART early during the treatment of an OI [18], [19]. However, in our study, subjects randomized to early ART were not more likely to develop IRIS; 6.3% of the subjects treated early experienced IRIS, compared to 10.4% of subjects who received deferred ART. Similar results were found in two prospective studies of cryptococcal IRIS, where earlier ART was not associated with the development of IRIS [10],[11].

A small randomized study conducted in Zimbabwe recently showed increased mortality with early ART in cryptococcal meningitis [20]. In this study, early ART was initiated within 72 hours of cryptococcal diagnosis. IRIS events were not evaluated, but the authors speculate that the increased mortality with early ART may have been due to increased rates of IRIS. If this is true, the increased rates of IRIS could have been due to ART being initiated very early (e.g., within 72 hours), as opposed to ART initiated a median of 12 days after the start of OI treatment, as in our study. The use of the more slowly fungicidal drug, fluconazole, in the Zimbabwe study, as opposed to amphotericin B in our study and others, could also account for the reported differences in the relationship between the timing of ART and IRIS in cryptococcal meningitis.

Furthermore, in the retrospective studies, residual confounding possibly related to unmeasured clinical factors or differences in adherence patterns could explain the association of earlier ART to IRIS [6], [17]. Indeed, in the Shelburne study, patients who developed IRIS appear to have been substantially more adherent to ART than their counterparts as evidenced by significantly higher rates of virologic suppression to <400 copies/mL at 24 months (78% vs. 36%; p<0.0001) [6]. If adherent patients were more likely to start treatment earlier (due to fewer social obstacles, greater motivation, more regular clinic attendance, etc.), this might account for the apparent relationship between earlier treatment initiation after acute OI presentation and the development of IRIS.

The receipt of corticosteroids during the management of the acute OI was not significantly associated with a reduction in the overall risk of IRIS, although a clinically meaningful reduction can not be excluded due to the relatively few patients who developed IRIS in this study. However, no patients developed IRIS while still on corticosteroids – thereby if not preventing at least possibly delaying the onset of IRIS. It is possible that a longer course of corticosteroids or other immune modulating agents could not only delay IRIS but actually reduce the risk of IRIS, but the risk/benefit of these types of approaches to IRIS would require systematic study. Also, it is possible that the lack of association between the receipt of corticosteroids and IRIS was due to confounding by indication, as IRIS has been associated with severity of underlying OI in some studies [5], [17], and subjects with more severe illness at baseline may have been more likely to have received corticosteroids.

In this study, in univariate analyses, baseline clinical features, other than the presenting OI (non-PCP fungal infections), did not distinguish subgroups at higher risk for IRIS. We did not find any significant differences in baseline characteristics between subjects who developed IRIS and those who did not, although the relatively small number of subjects who developed IRIS in the study may have limited our ability to detect differences.

However, in multivariate time-varying Cox models, after controlling for immune and virologic parameters over time and non-PCP fungal infections, baseline CD4+ T-cell count, CD4+ percentage, and HIV RNA levels were predictors of IRIS, consistent with previous research [7], [12], [21]. The apparent incongruity between the findings in the univariate and Cox analyses regarding the importance of these parameters in the prediction of IRIS implies complex relationships between baseline immunologic and virologic values, these parameters over time, and the development of IRIS (e.g., those with lower baseline CD4+ T-cell counts or higher HIV RNA levels were both more likely to have a more robust response to ART and to develop IRIS). Perhaps, because of relatively uniform low pre-ART CD4+ T-cell counts and high HIV RNA levels, the significance of baseline values only emerges in models that jointly consider baseline and values over time. Like others, we found higher CD4+ T-cell counts and CD4+ percentages and lower HIV RNA levels on treatment to be associated with the development of IRIS [5], [10], [21].

Patients with known TB were excluded from the trial, and it is unknown whether these results would be applicable to individuals presenting with TB. Furthermore, ACTG A5164 enrolled predominantly subjects with PCP, and corticosteroids were used frequently during the study. There is limited power to generalize the conclusions of this study to less common OIs and to patients who are not given corticosteroids during the management of their acute OI. However, ACTG A5164 is the largest trial to date which has reported the effects of the timing of ART during an acute OI on the rates of IRIS, and there was no trend towards increased IRIS with early ART for any entry OI or in subjects who did not receive corticosteroids for their acute OI. Due to the multiple comparisons involved in this study, marginally significant associations should be interpreted cautiously. Also, case reviewers were limited by what was prospectively recorded by the site. Data on severity of the presenting OI were not uniformly available while information on symptoms of IRIS, medications, and laboratory data were more complete.

In conclusion, retrospective reports appear to have overestimated the occurrence of IRIS after ART initiation in advanced HIV disease. The presence of low baseline CD4+ T-cell count and high HIV RNA levels, a non-PCP fungal infection, and improved immunologic and virologic response to ART predict the development of IRIS. Corticosteroids, as used in this study, may delay the onset of IRIS but were not associated with a reduction in its frequency. In subjects with non-TB OIs, early initiation of ART does not increase the incidence of IRIS, and concern about IRIS should not be a reason to defer ART.

## Methods

The protocol for the primary study and supporting CONSORT checklist are available as supporting information; see Checklist S1 and Protocol S1.

### Ethics Statement

This study was conducted according to the principles expressed in the Declaration of Helsinki. The protocol was approved by all 46 of the participating sites' Institutional Review Boards, including the Stanford University Institutional Review Board, the lead site for the study. Written informed consent was obtained from subjects prior to entry.

AIDS Clinical Trials Group (ACTG) A5164 was a randomized trial of early versus deferred ART in subjects with an acute AIDS-defining OI or a serious bacterial infection (defined as bacterial pneumonia or other bacterial infection of a deep tissue, body cavity, or other normally sterile site and a CD4+ T-cell count<200 cells/µL). Allowable entry OIs included *Pneumocystis jirovecii* pneumonia (PCP), other fungal infections (including those due to cryptococcus or histoplasma), toxoplasmosis, cytomegalovirus infection, and non-tuberculous mycobacterial infection. Patients with tuberculosis (TB) were excluded from the study but were allowed to stay on-study if the diagnosis was made after randomization. Subjects were only allowed to have minimal ART exposure prior to study entry (no ART within 8 weeks prior to study entry, no more than 31 days of any ART within 6 months prior to study entry, and no more than one ART regimen on which they had experienced treatment failure). Subjects were enrolled from the United States, including Puerto Rico, and South Africa. Subjects were randomized within 14 days of starting therapy for the OI (or bacterial infection) that determined study eligibility. Subjects were randomized 1∶1 to receive ART immediately after study entry or to have ART deferred for at least 4 weeks after randomization. The study provided lopinavir/ritonavir, stavudine, and, starting in September 2005, tenofovir/emtricitabine, but clinicians could select any standard, recommended ART regimen.

Subjects were followed prospectively with study visits at weeks 4, 8, 12, 16, 24, 32, 40, and 48, and at time of suspected IRIS, if outside the scheduled visit window. Measurement of CD4+ and CD8+ T cells and plasma HIV RNA levels were performed at study entry, at initiation of ART, at weeks 4, 8, 12, 16, 24, 32, and 40 following initiation of ART, at time of suspected IRIS, and at study completion. Further details of the trial have been reported elsewhere [13].

IRIS was pre-defined in the protocol as symptoms consistent with an infectious/inflammatory condition, temporally related to the initiation of ART and associated with an increase in CD4+ T-cell count and/or a decrease in plasma HIV-1 RNA levels, but not explained by a newly acquired infection, the expected clinical course of a previously diagnosed infection, or the side effects of ART. When IRIS was diagnosed by a site investigator, case records were reviewed by a study chair (W.P. or A.Z.) or an independent reviewer (all blinded to study arm assignment) for confirmation. To confirm that sites had not under-reported IRIS, an independent reviewer retrospectively evaluated study records of all subjects who were prescribed corticosteroids or non-steroidal anti-inflammatory drugs (NSAIDs) during the study in an attempt to uncover additional cases of IRIS. Details of the clinical presentation, management, and outcome of IRIS were extracted from study records by two investigators (P.G. and A.Z.) using a standardized abstraction tool.

As the study definition of IRIS is only applicable to subjects who started ART, this analysis is limited to subjects who initiated ART and had at least one subsequent study visit with baseline defined as the time of ART initiation. Comparisons between subjects with and without an IRIS diagnosis were evaluated using the Wilcoxon rank-sum test for continuous and ordered categorical variables and Fisher's exact test for categorical variables. P-values<0.05 were considered statistically significant. In this secondary analysis, results were not adjusted for multiple comparisons. Logistic regression was used to investigate the association between baseline characteristics, laboratory values and entry OIs with the odds of having an IRIS diagnosis. Cox models with time-varying covariates were used to evaluate the association between IRIS diagnosis and CD4+ and CD8+ T-cell counts, CD4+ and CD8+ percentages, and HIV RNA levels prior to IRIS diagnosis. Time-varying covariates used a last value carried forward approach until the time a new measurement was obtained.

## Supporting Information

Checklist S1 CONSORT Checklist
(0.06 MB DOC)

Protocol S1 Trial Protocol
(0.58 MB DOC)
